# Deletion of Aldose Reductase from Mice Inhibits Diabetes-Induced Retinal Capillary Degeneration and Superoxide Generation

**DOI:** 10.1371/journal.pone.0062081

**Published:** 2013-04-16

**Authors:** Jie Tang, Yunpeng Du, J. Mark Petrash, Nader Sheibani, Timothy S. Kern

**Affiliations:** 1 Department of Medicine, Case Western Reserve University, Cleveland, Ohio, United States of America; 2 Department of Ophthalmology, Heilongjiang Province Hospital, Heilongjiang Province, Harbin, China; 3 Department of Ophthalmology, University of Colorado, Denver, Colorado, United States of America; 4 Department of Ophthalmology, University of Wisconsin, Madison, Wisconsin, United States of America; 5 Cleveland VAMC Research Service 151, Cleveland, Ohio, United States of America; University of Florida, United States of America

## Abstract

**Purpose:**

Pharmacologic inhibition of aldose reductase (AR) previously has been studied with respect to diabetic retinopathy with mixed results. Since drugs can have off-target effects, we studied the effects of AR deletion on the development and molecular abnormalities that contribute to diabetic retinopathy. Since recent data suggests an important role for leukocytes in the development of the retinopathy, we determined also if AR in leukocytes contributes to leukocyte-mediated death of retinal endothelial cells in diabetes.

**Methods:**

Wild-type (WT; C57BL/6J) and AR deficient (AR^−/−^) mice were made diabetic with streptozotocin. Mice were sacrificed at 2 and 10 months of diabetes to evaluate retinal vascular histopathology, to quantify retinal superoxide production and biochemical and physiological abnormalities in the retina, and to assess the number of retinal endothelial cells killed by blood leukocytes in a co-culture system.

**Results:**

Diabetes in WT mice developed the expected degeneration of retinal capillaries, and increased generation of superoxide by the retina. Leukocytes from diabetic WT mice also killed more retinal endothelial cells than did leukocytes from nondiabetic animals (p<0.0001). Deletion of AR largely (P<0.05) inhibited the diabetes-induced degeneration of retinal capillaries, as well as the increase in superoxide production by retina. AR-deficiency significantly inhibited the diabetes-induced increase in expression of inducible nitric oxide synthase (iNOS) in retina, but had no significant effect on expression of intercellular adhesion molecule-1 (ICAM-1), phosphorylated p38 MAPK, or killing of retinal endothelial cells by leukocytes.

**Conclusions:**

AR contributes to the degeneration of retinal capillaries in diabetic mice. Deletion of the enzyme inhibits the diabetes-induced increase in expression of iNOS and of superoxide production, but does not correct a variety of other pro-inflammatory abnormalities associated with the development of diabetic retinopathy.

## Introduction

Diabetic retinopathy is a common complication of diabetes, and is the principal cause of blindness in working-aged adults. Hyperglycemia clearly initiates the disease process, but which of the sequelae of hyperglycemia are causal in development of the retinopathy is not clear.

Activation of aldose reductase (AR) by elevated glucose was one of the first biochemical mechanisms postulated to explain the pathogenesis of diabetic complications [1]. Particularly with regard to cataractogenesis, evidence suggested that pathology occurred as a result of osmotic consequences of AR-mediated reduction of glucose to its polyol, sorbitol [2]. Since then, AR has been found also to regulate a variety of additional abnormalities (including oxidative stress and inflammation) that have been implicated in the pathogenesis of various complications of diabetes [3]–[5] and other diseases [6]–[16].

Efforts to inhibit this pathway to inhibit diabetic retinopathy in patients have relied heavily on pharmacologic inhibitors of the enzyme [17]. Nevertheless, results of these studies with regard to the retinopathy have been inconsistent and controversial. Some studies of diabetic or galactosemic rodents or dogs showed beneficial effects of AR inhibitors on lesions of the retinpathy [18]–[23], whereas no significant effects were detected in clinical studies in patients and other studies of diabetic and galactosemic dogs and rodents [24]–[27]. Possibilities that have been discussed to explain the lack of agreement among studies include differences in the degree of inhibition of AR, the presence of isoforms of the enzyme that respond differently to therapies, genetic differences among individuals, and possible off-target effects of the different AR inhibitors [17].

Generation of animals that are totally deficient in AR is one way to overcome the shortcomings associated with the use of pharmacologic inhibitors of AR. Moreover, AR^−/−^ animals allow the opportunity to investigate the molecular pathways by which AR acts in hyperglycemia. Thus, the availability of AR^−/−^ animals makes it worthwhile to revisit this topic. In the present study, we investigated the effect of AR deficiency on diabetes-induced degeneration of retinal capillaries in early diabetic retinopathy, and on diabetes-induced pro-inflammatory and pro-oxidant changes in the retina.

## Research Design and Methods

### Experimental Animals

AR^−/−^ mice were prepared [28] and backcrossed with C57BL/6 mice for seven generations. Male C57Bl/6J mice and AR^−/−^ mice were randomly assigned to become diabetic or remain as nondiabetic group. Diabetes was induced by 5 sequential daily intraperitoneal injections of a freshly prepared solution of streptozotocin in citrate buffer (pH 4.5) at 45 mg/kg of body weight. Insulin was given as needed to prevent weight loss without preventing hyperglycemia and glucosuria (0–0.2 units of NPH insulin subcutaneously, 0–3 times per week). Glycohemoglobin (GHb) was measured by Bio-Rad Total Glycated Hemoglobin Assay (Bio-Rad Laboratories, Inc, Hercules, CA, USA) every 2–3 mos and just before animals were sacrificed. Food consumption and body weight were measured weekly. Animals were studied for durations of 10 months or 2 months of diabetes in order to investigate effects of the therapy on retinal histopathology, or molecular and physiologic changes, respectively. These durations were chosen because 2 mos diabetes has been found to result in numerous metabolic alterations which precede (and likely contribute to) the later appearance of vascular histopathology, and 10 mos duration of diabetes has been shown to have developed robust vascular histopathology characteristic of the early stages of the retinopathy.

### Ethics Statement

Treatment of animals conformed to the ARVO Resolution on Treatment of Animals in Research, as well as to institutional guidelines (Case Western Reserve University IACUC # 2010–0156).

#### Retinal histopathology

After 10 months of diabetes, one retina was fixed in 10% buffered formalin, washed in running water overnight, and digested in elastase (Calbiochem, Cat#324682) buffer (40 unit/ml elastase in 100 mM sodium phosphate buffer (containing 150 mM sodium chloride and 5 mM ethylenediamene tetraacetic acid (EDTA; pH 6.5) in an agitating water bath at 37° for 1½ h [29]. Then, retinas were washed overnight in 100 mM Tris-HCI (pH 8.5) at room temperature, and cleaning was finished the next day. When totally cleaned of neural cells, the isolated vasculature was laid out on a glass microscope slide, dried overnight, stained with hematoxylin and periodic acid-Schiff, dehydrated and coverslipped. Degenerate (acellular) capillaries were quantitated in 6–7 field areas corresponding to the mid-retina in a masked manner. Acellular capillaries were identified as capillary-sized vessel tubes having no nuclei anywhere along their length, and were reported per square millimeter of retinal area.

### Superoxide Measurement

At 2 months of diabetes, fresh retinas from animals were analyzed for superoxide production as previously described [30], [31]. Briefly, whole retinas were placed in 0.2 ml Krebs/Hepes buffer and allowed to equilibrate in the dark for 30 mins at 37°C under 5% CO_2_. To each tube, 0.5 mM lucigenin (Sigma Chemical Company, St. Louis, MO) was added, and photon emission was detected over the following 10 min using a luminometer. Retinal protein was quantified per samples (Bio-Rad Laboratories, Inc, Hercules, CA, USA) and luminescence was expressed per milligram protein.

### Western Blots

At 2 months of diabetes, retinal homogenates separated by SDS-PAGE, and transferred to nitrocellulose membrane (Bio-Rad Laboratories, Inc, Hercules, CA, USA). Membranes were blocked in Tris-buffered saline containing 0.02% Tween 20 and 5% nonfat milk., washed, and were incubated with anti-rat ICAM-1 (1∶2000 dilution; R&D Systems, Minneapolis, MN), anti-rat iNOS (1∶1000 dilution; Santa Cruz Biotechnology, Inc, Santa Cruz, CA), anti-rat nitrotyrosine (1;1000 dilution; Upstate Biotechnology, Inc., Billerca, MA), anti-rat p38 MAPK and p-p38 MAPK (1;1000 dilution; Cell Signaling Technology Inc., Danvers, MA) for 2 hrs, and then stained with respective horseradish peroxidase coupled secondary antibody (Bio-Rad Laboratories, Inc, Hercules, CA) at a dilution of 1∶3000 for 1 hr. After extensive washing, immunostaining detected by the antibodies was visualized by enhanced chemiluminescence (ECL, Santa Cruz Biotechnology, Santa Cruz, CA). The protein levels were quantitated relative to ß-actin-loading control (1∶3000 dilution, Abcam, Inc., Cambridge, MA) in the same samples. Results are expressed as a percent of values detected in the nondiabetic controls. Protein was quantified with the Bio-Rad protein assay (Bio-Rad Laboratories, Inc; Hercules, CA).

### Reduced glutathione (GSH)

At 2 months of diabetes, tissues of interest were homogenized in 0.1 M phosphate buffer (pH 8.0) containing 5 mM EDTA. After centrifugation, a quantity of the resulting supernatant was removed for storage at −80°C for subsequent protein determination by the bicinchoninic acid protein assay method (Pierce Biotechnology, Inc.). GSH determinations were carried out on the freshly prepared tissue extract following the fluorometric method described previously [32]. Fluorometric measurements were carried out using a Biotek Synergy4 microplate reader and data fitted by linear regression to a standard curve generated using purified GSH as a quantitation standard. In all cases, analytes were measured in triplicate.

### Co-culture

A mouse retinal endothelial cell line (mREC; generated from Immortomice [33]) was grown in DMEM containing 10% FBS and 5.5 or 25 mM glucose. Cells were cultured at 37°C in 5% CO_2_ and 95% air, and the media was changed every other day for 5 days. When mRECs were 80% confluent (app 500,000 cells), leukocytes (100,000; purified from blood with RBC lysis buffer) from male C57Bl/6J mice and AR^−/−^ mice groups were added to the mREC and incubated for 24 hrs. After incubation, the leukocytes were carefully removed by gentle washing, and viability of remaining retinal endothelial cells was measured by trypan blue extrusion. Briefly, an aliquot of the endothelial cell suspension was diluted 1∶1 (vol/vol) with 0.1% trypan blue, and the cells were counted with a hemocytometer. Cell death was reported as the percentage of blue-stained cells (dead cells) of the total number of cells. Approximately 200–400 cells were counted in each sample.

### Statistical analyses

Data are expressed as mean ± SD. Statistical analysis was performed using ANOVA, followed by Fischer's test. A value of p<0.05 was considered statistically significant.

## Results

### Animals

Diabetic mice were hyperglycemic over the entire duration of the 10 month experiment, and failed to gain weight at normal rate. Serum glucose levels of the diabetic groups (356±58 mg/dl and 344±62 in in wildtype and AR^−/−^ groups, respectively) were all significantly greater than nondiabetic values (132–137 mg/dl). The degree of hyperglycemia, as denoted by glycated hemoglobin, did not vary among diabetic mice (wild-type, 10.1%±1.0 ; AR^−/−^, 10.2%±1.1 respectively), and was significantly greater in diabetic groups as compared with the corresponding nondiabetic groups (wildtype, 3.0±0.2; AR^−/−^ 2.9±0.3). Body weight at 10 months of diabetes averaged 28 g±1in the wildtype group and 27 g±2 in AR^−/−^ group, and these values were about half of those in nondiabetic mice of each group (51–60 g). Data from the 2 month experiment was similar (not shown). Deletion of AR had no apparent effect on glycemia or health of any animals. Cross-sections of retina in nondiabetic animals (12 mos of age) indicated that deletion of AR had no significant effects on retinal thickness or retinal architecture, and no effect on vascular density in nondiabetic animals.

### Inhibition of diabetes-induced retinal histopathology by AR deletion

**Figure 1:**
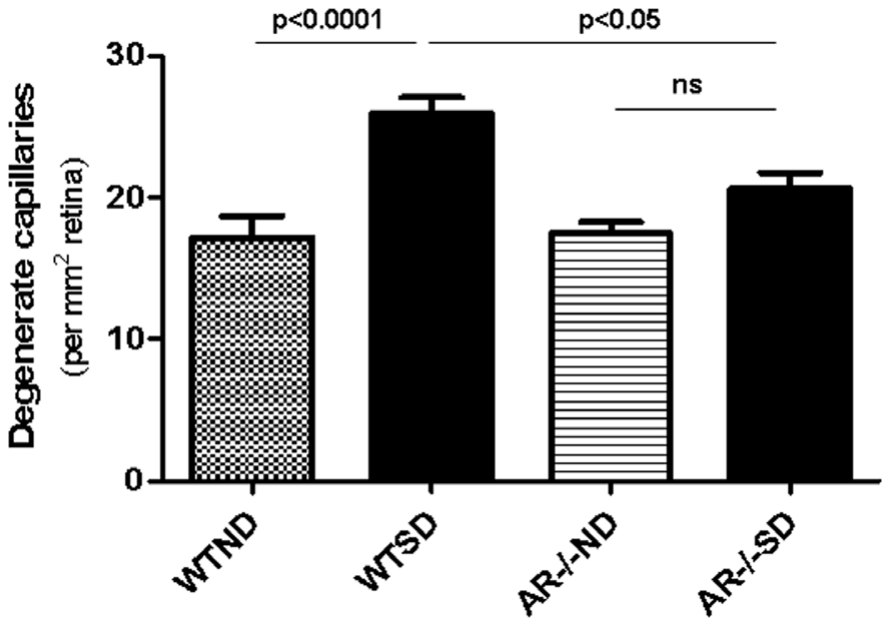
Diabetes (SD) of 10 months duration significantly increased degeneration of retinal capillaries in wildtype (WT) animals compared to nondiabetic (ND) animals. Deletion of AR significantly, but only partially, inhibited the diabetes-induced vascular degeneration (n = 7 per group).

Wild-type diabetics developed the expected increase in numbers of degenerate retinal capillaries by 10 mos of study (p<0.0001). In contrast, retinas from AR^−/−^ diabetic animals exhibited significantly fewer diabetes-induced acellular capillaries (Figure 1). Nondiabetic AR^−/−^ mice showed a normal retinal vasculature pattern. Diabetes of 10 mos duration did not cause a significant decrease in the number of cells in the retinal ganglion cell layer in these C57Bl/6 mice (not shown), so we could not assess any effect of the AR deletion on neurodegeneration.

In an effort to investigate the mechanism by which the AR deletion mediated the beneficial effect on degeneration of retinal capillaries in diabetes, we measured several parameters related especially to inflammation and nitrative (oxidative) stress, these being physiologic and molecular abnormalities that have been found in other studies to be associated with (and possibly causally related to) the development of the early stages of diabetic retinopathy.

### Oxidative stress

**Figure 2:**
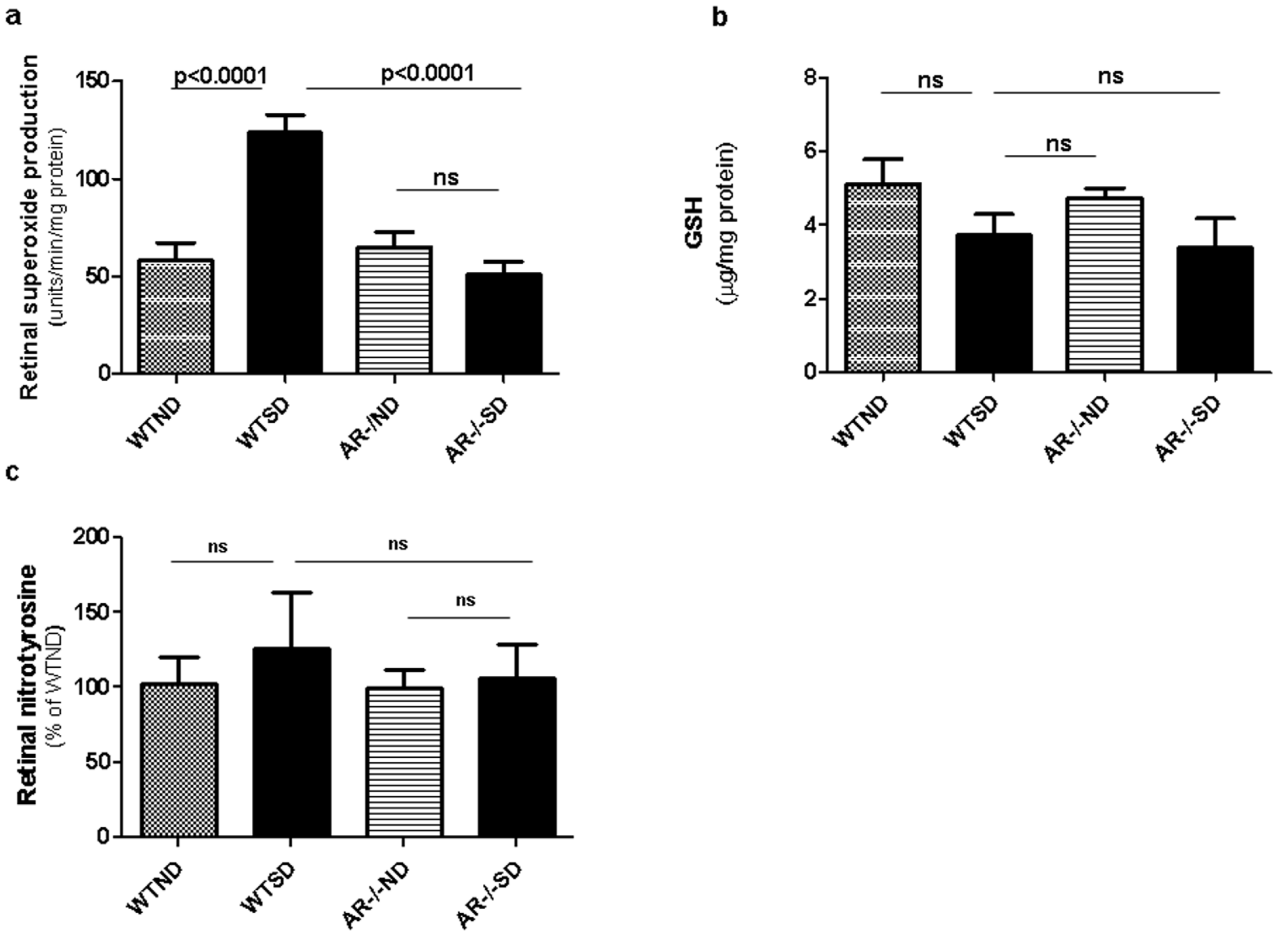
Effects of 2 mos diabetes and AR−/− on retinal oxidative stress. (a) Retinas from diabetic WT mice generated significantly more superoxide than nondiabetic WT mice, and the diabetes-induced superoxide generation was inhibited by deletion of AR. Reduced glutathione (GSH) (b) tended to become subnormal, and nitrotyrosine (c) tended to become increased in diabetes, but these changes were not statistically significant, and were not corrected in AR−/− mice. n = 4–6 per experimental group.

Oxidative stress was evaluated in the present study by measuring superoxide generation by the freshly isolated retina. Diabetes significantly increased retinal superoxide production by almost two-fold (Figure 2a), whereas retinas from AR^−/−^ mice were totally protected from diabetes-induced increase in superoxide production (p<0.0001). In contrast, retinal GSH tended to be subnormal in wild-type diabetics, but deletion of AR had little effect on this diabetes-induced reduction (Figure 2b). The accumulation of nitrotyrosine, a marker of nitrative stress, tended to be greater than normal in wild-type diabetic animals and tended to be inhibited in the absence of AR, but the results were not statistically significant (Figure 2c).

### Pro- inflammatory proteins

**Figure 3:**
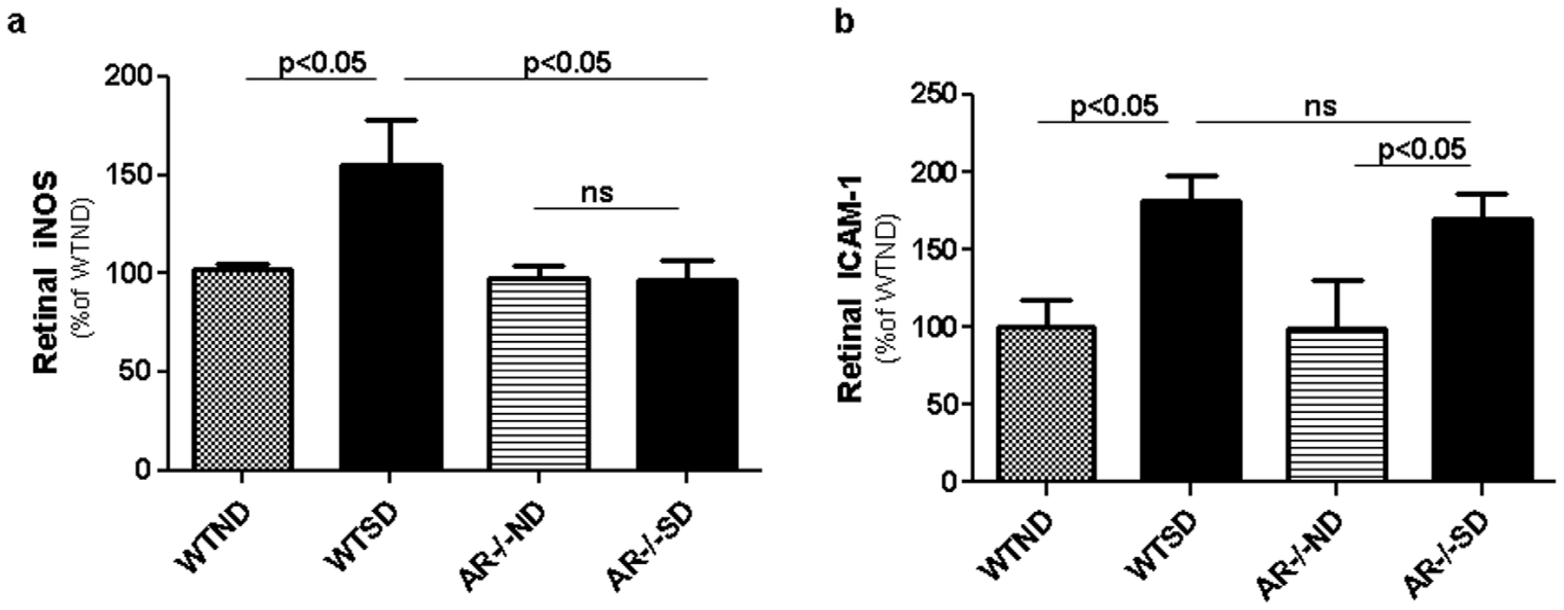
Diabetes (2 month duration) increased expression of (a) iNOS and (b) ICAM-1 in retina. Deletion of AR inhibited the diabetes-induced increase in iNOS, but had no effect on ICAM-1 expression. n = 4–6 per experimental group.

Expression of iNOS and ICAM-1 in whole retina were significantly increased by diabetes compared to that of wildtype nondiabetic animals (both p<0.05), but deletion of AR inhibited the diabetes-induced enzyme induction only for iNOS (Figure 3). The expression of phosphorylated p38 MAPK, and activity of poly-ADP ribose synthase (PARS) tended to be greater than normal in wild-type diabetic animals, but the increases were not statistical significant and deletion of AR had no apparent effect (not shown).

### Endothelial cells killed by white blood cells

**Figure 4:**
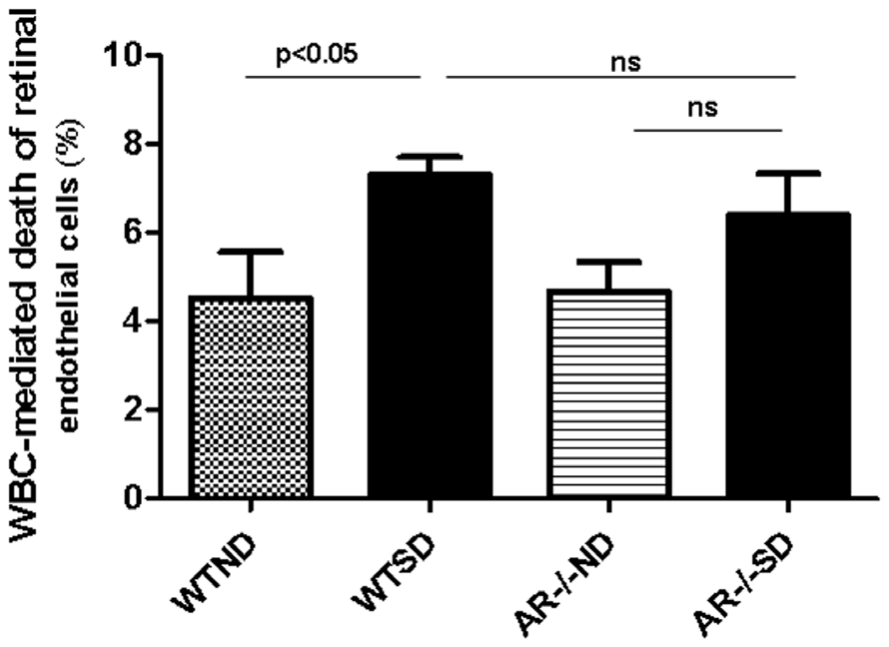
Leukocytes from diabetic WT mice killed significantly more retinal endothelial cells than did leukocytes from nondiabetic mice. Deletion of AR did not significantly inhibit this abnormality. Leukocytes were collected from 3 animals per group for co-culture studies.

Previous studies have demonstrated an important role of leukocytes in development of early stages of diabetic retinopathy [34], [35]. To investigate if white blood cells might contribute to the AR-mediated degeneration of retinal capillaries in diabetes, mouse retinal endothelial cells (mREC) were incubated with peripheral blood leukocytes from non-diabetic or diabetic mice. After 24 hrs of co-culture, the number of endothelial cells killed by leukocytes from wild-type diabetic mice was significantly greater than those killed by incubation with leukocytes from nondiabetic wild-type mice (1.7 fold increase; p<0.05; Figure 4). In contrast, leukocytes from diabetic animals lacking AR killed fewer endothelial cells than did leukocytes from wild-type diabetics, but this decrease did not achieve statistical significance.

## Discussion

AR and the polyol pathway were among the first molecular mechanisms considered to explain how hyperglycemia initiates diabetic retinopathy and other complications of diabetes. At that time, AR was regarded as the enzyme responsible for the reduction of glucose into sorbitol, a molecule that penetrates poorly through cell membranes, thus leading to cellular damage via the “osmotic hypothesis” [2]. In an effort to further investigate the role of AR in these complications, a number of pharmacologic inhibitors of the enzyme were developed. Since then, research on this pathway has expanded beyond the initial hypothesis, but results using pharmacologic inhibitors of AR in clinical (and some pre-clinical) studies of diabetic retinopathy have been disappointing. Whether this was due to inadequacies of the pharmacologic inhibitors, limited penetration of target tissues, or unfavorable pharmacokinetic characteristics has remained unclear.

Use of AR inhibitors revealed a variety of diabetes-induced alterations in retinal metabolism that could be manipulated. AR inhibitors were reported to inhibit diabetes-induced abnormalities in oxidative stress, inflammation, and apoptosis in the retina [5], [36], [37], [38], [39]. Recent studies using pharmacologic inhibitors of AR found anti-inflammatory actions even in the absence of hyperglycemia [3], [6], [11]–[16], [40]–[43]. Thus, it is unclear whether the observed effects of AR inhibitors are really mediated via hyperglycemia-induced activation of polyol pathway activity or alternatively via other mechanisms possibly unrelated to glucose metabolism (such as metabolism of lipid-derived aldehydes and retinoids by AR and closely-related aldo-keto reductases [44], [45]).

To avoid potential uncertainties associated with pharmacologic inhibitors, mice genetically lacking AR were generated [28], [46]. Initial studies of db/db mice deficient in AR suggested that AR contributed to the diabetes-induced abnormalities in the retina, including breakdown of the blood-retinal barrier, loss of pericytes, and even an effect on neovascularization was claimed. The present results using C57Bl/6 mice that are deficient in AR demonstrated that this genetic modification substantially (but not totally) inhibited the diabetes-induced degeneration of retinal capillaries, and totally inhibited superoxide generation by the retina in diabetes. Consistent with most other studies of diabetic mice, however, no retinal neovascularization was observed. Unlike studies using pharmacologic inhibitor, genetic deletion of AR had no significant effect on retinal GSH levels in diabetes in the present study. Consistent with this, reported inhibition of diabetes-induced defects in electroretinogram using pharmacologic inhibitors [47]–[49] was not confirmed using AR^−/−^ mice studied by us [50], raising a possibility that some of the observed effects of AR inhibitors were due to off-target effects. In addition, the differences could be secondary to metabolic pathways induced in response to the deletion of AR, thus altering the retinal environment. Deletion of AR also inhibited the diabetes-induced increase in expression of iNOS but not ICAM-1, which is interesting because both are regulated by NF-*κ*B, and both have been shown to be strongly related to the capillary degeneration occurring in retinas of diabetic animals [51], [52]. Inasmuch as superoxide and iNOS-generated NO can lead to the generation of nitrotyrosine on proteins [53], [54], it is surprising that AR deletion inhibited both superoxide and iNOS expression in diabetic mice, yet did not correct nitrotyrosine. This finding needs to be confirmed with additional animals.

It has been assumed by many that the molecular abnormalities contributing to vascular pathology in diabetic retinopathy are present in vascular cells themselves. In vitro studies have indicated effects of AR within endothelial cells [35,54,55. Recent evidence indicating that leukocytes play a critical role in the capillary degeneration [Li, 2012 #6144], however, led us to test the possibility that AR present in leukocytes might contribute to the capillary cell death. Our in vitro studies compared leukocyte-mediated killing of retinal endothelial cells by leukocytes having, and not having, AR. These results suggest that AR contributes only little to the hyperglycemia-induced killing of endothelial cells by leukocytes, suggesting that the beneficial effect of AR deletion might in fact be within cells of the retina itself.

Our results utilizing AR mutant mice, together with results from other investigators using a similar approach [46], demonstrate that AR contributes to the pathogenesis of vascular pathology of early diabetic retinal disease in mice. Surprisingly, the AR knockout did not inhibit several diabetes-induced molecular abnormalities that previously have been found to contribute to the retinopathy [51], [56], suggesting that multiple molecular pathways contribute to the vascular lesions of the retinopathy in mice. Perhaps the relative contributions of these various pathways differs among species, and these differences among species contributed to the lack of significant effect of AR inhibitors in diabetic dogs [25] or patients [24], in contrast to more positive effects in rodents. In the absence of information as to which pathways predominate in the development of retinopathy in diabetic humans, future strategies probably will benefit from targeting of multiple pathways that each partially contribute to the pathogenesis of the retinopathy.

## References

[1] Kinoshita JH (1986) Aldose reductase in the diabetic eye. Am J Ophthalmol 102: 685–692. 3098107 10.1016/0002-9394(86)90394-6

[2] Kinoshita JH (1990) A thirty year journey in the polyol pathway. Exp Eye Res 50: 567–573. 2115448 10.1016/0014-4835(90)90096-d

[3] Ramana KV, Bhatnagar A, Srivastava SK (2004) Inhibition of aldose reductase attenuates TNF-alpha-induced expression of adhesion molecules in endothelial cells. FASEB J 18: 1209–1218. 15284221 10.1096/fj.04-1650com

[4] Ramana KV, Friedrich B, Srivastava S, Bhatnagar A, Srivastava SK (2004) Activation of nuclear factor-kappaB by hyperglycemia in vascular smooth muscle cells is regulated by aldose reductase. Diabetes 53: 2910–2920. 15504972 10.2337/diabetes.53.11.2910

[5] Obrosova IG, Pacher P, Szabo C, Zsengeller Z, Hirooka H, et al (2005) Aldose reductase inhibition counteracts oxidative-nitrosative stress and poly(ADP-ribose) polymerase activation in tissue sites for diabetes complications. Diabetes 54: 234–242. 15616034 10.2337/diabetes.54.1.234 PMC2756473

[6] Pladzyk A, Reddy AB, Yadav UC, Tammali R, Ramana KV, et al (2006) Inhibition of aldose reductase prevents lipopolysaccharide-induced inflammatory response in human lens epithelial cells. Invest Ophthalmol Vis Sci 47: 5395–5403. 17122129 10.1167/iovs.06-0469

[7] Ramana KV, Willis MS, White MD, Horton JW, DiMaio JM, et al (2006) Endotoxin-induced cardiomyopathy and systemic inflammation in mice is prevented by aldose reductase inhibition. Circulation 114: 1838–1846. 17030682 10.1161/CIRCULATIONAHA.106.630830

[8] Tammali R, Ramana KV, Singhal SS, Awasthi S, Srivastava SK (2006) Aldose reductase regulates growth factor-induced cyclooxygenase-2 expression and prostaglandin e2 production in human colon cancer cells. Cancer Res 66: 9705–9713. 17018629 10.1158/0008-5472.CAN-06-2105

[9] Cheung AK, Lo AC, So KF, Chung SS, Chung SK (2007) Gene deletion and pharmacological inhibition of aldose reductase protect against retinal ischemic injury. Exp Eye Res 85: 608–616. 17727843 10.1016/j.exer.2007.07.013

[10] Lo AC, Cheung AK, Hung VK, Yeung CM, He QY, et al (2007) Deletion of aldose reductase leads to protection against cerebral ischemic injury. J Cereb Blood Flow Metab 27: 1496–1509. 17293845 10.1038/sj.jcbfm.9600452

[11] Yadav UC, Srivastava SK, Ramana KV (2007) Aldose reductase inhibition prevents endotoxin-induced uveitis in rats. Invest Ophthalmol Vis Sci 48: 4634–4642. 17898287 10.1167/iovs.07-0485 PMC2377062

[12] Reddy AB, Srivastava SK, Ramana KV (2009) Anti-inflammatory effect of aldose reductase inhibition in murine polymicrobial sepsis. Cytokine 48: 170–176. 19660963 10.1016/j.cyto.2009.07.004 PMC2767443

[13] Ramana KV, Srivastava SK (2010) Aldose reductase: a novel therapeutic target for inflammatory pathologies. Int J Biochem Cell Biol 42: 17–20. 19778627 10.1016/j.biocel.2009.09.009 PMC2787653

[14] Yadav UC, Srivastava SK, Ramana KV (2010) Understanding the role of aldose reductase in ocular inflammation. Curr Mol Med 10: 540–549. 20642441 10.2174/1566524011009060540 PMC2912437

[15] Srivastava SK, Yadav UC, Reddy AB, Saxena A, Tammali R, et al (2011) Aldose reductase inhibition suppresses oxidative stress-induced inflammatory disorders. Chem Biol Interact 191: 330–338. 21354119 10.1016/j.cbi.2011.02.023 PMC3103634

[16] Yadav UC, Ramana KV, Srivastava SK (2011) Aldose reductase inhibition suppresses airway inflammation. Chem Biol Interact 191: 339–345. 21334316 10.1016/j.cbi.2011.02.014 PMC3103624

[17] Chung SS, Chung SK (2005) Aldose reductase in diabetic microvascular complications. Curr Drug Targets 6: 475–486. 16026266 10.2174/1389450054021891

[18] Robison WG Jr, Kador PF, Akagi Y, Kinoshita JH, Gonzalez R, et al (1986) Prevention of basement membrane thickening in retinal capillaries by a novel inhibitor of aldose reductase, tolrestat. Diabetes 35: 295–299. 3081393 10.2337/diab.35.3.295

[19] Kador PF, Akagi Y, Terubayashi H, Wyman M, Kinoshita JH (1988) Prevention of pericyte ghost formation in retinal capillaries of galactose-fed dogs by aldose reductase inhibitors. Arch Ophthalmol 106: 1099–1102. 3401138 10.1001/archopht.1988.01060140255036

[20] Robison WG Jr, Nagata M, Laver N, Hohman TC, Kinoshita JH (1989) Diabetic-like retinopathy in rats prevented with an aldose reductase inhibitor. Invest Ophthalmol Vis Sci 30: 2285–2292. 2509395

[21] Akagi Y, Kador PF (1990) Effect of aldose reductase inhibitors on the progression of retinopathy in galactose-fed dogs. Exp Eye Res 50: 635–639. 2115453 10.1016/0014-4835(90)90106-5

[22] Robison WG Jr, Tillis TN, Laver N, Kinoshita JH (1990) Diabetes-related histopathologies of the rat retina prevented with an aldose reductase inhibitor. Exp Eye Res 50: 355–366. 2110907 10.1016/0014-4835(90)90136-i

[23] Kador PF, Takahashi Y, Sato S, Wyman M (1991) Aldose reductase, retinal vessel changes and cataracts in galactose-fed dogs. In: Rifkin H, Colwell JA, Taylor SI, editors. Diabetes 1991. Amsterdam: Elsevier Science Publishers. pp. 373–378.

[24] Sorbinil Retinopathy Trial Research Group (1990) A randomized trial of sorbinil, an aldose reductase inhibitor, in diabetic retinopathy. Arch Ophthalmol 108: 1234–1244. 2119168 10.1001/archopht.1990.01070110050024

[25] Engerman RL, Kern TS (1993) Aldose reductase inhibition fails to prevent retinopathy in diabetic and galactosemic dogs. Diabetes 42: 820–825. 8495805 10.2337/diab.42.6.820

[26] Engerman RL, Kern TS, Garment MB (1993) Capillary basement membrane in retina, kidney, and muscle of diabetic dogs and galactosemic dogs and its response to 5 years aldose reductase inhibition. J Diab Compl 7: 241–245. 8219367

[27] Kern TS, Engerman RL (1995) Galactose-induced retinal microangiopathy in rats. Invest Ophthalmol Vis Sci 36: 490–496. 7843917

[28] Ho HT, Chung SK, Law JW, Ko BC, Tam SC, et al (2000) Aldose reductase-deficient mice develop nephrogenic diabetes insipidus. Mol Cell Biol 20: 5840–5846. 10913167 10.1128/mcb.20.16.5840-5846.2000 PMC86061

[29] Laver NM, Robison WG Jr, Pfeffer BA (1993) Novel procedures for isolating intact retinal vascular beds from diabetic humans and animal models. Invest Ophthalmol Vis Sci 34: 2097–2104. 8491560

[30] Du Y, Miller CM, Kern TS (2003) Hyperglycemia increases mitochondrial superoxide in retina and retinal cells. Free Radic Biol Med 35: 1491–1499. 14642397 10.1016/j.freeradbiomed.2003.08.018

[31] Li G, Tang J, Du Y, Lee CA, Kern TS (2011) Beneficial effects of RAGE-Ig fusion protein on early diabetic retinopathy and tactile allodynia. Molecular Vision 17: 3156–3165. 22171162 PMC3235538

[32] Cohn VH, Lyle J (1966) A fluorometric assay for glutathione. Anal Biochem 14: 434–440. 5944947 10.1016/0003-2697(66)90286-7

[33] Su X, Sorenson CM, Sheibani N (2003) Isolation and characterization of murine retinal endothelial cells. Mol Vis 9: 171–178. 12740568

[34] Li G, Veenstra AA, Talahalli RR, Wang X, Gubitosi-Klug RA, et al.. (2012) Marrow-Derived Cells Regulate the Development of Early Diabetic Retinopathy and Tactile Allodynia in Mice. Diabetes. 10.2337/db11-1249 PMC3501859 22923475

[35] Talahalli R, Zarini S, Tang J, Li G, Murphy R, et al.. (2012) Leukocytes regulate retinal capillary degeneration in the diabetic mouse via generation of leukotrienes. J Leukoc Biol. 10.1189/jlb.0112025 PMC3525833 23108096

[36] Obrosova IG, Minchenko AG, Vasupuram R, White L, Abatan OI, et al (2003) Aldose reductase inhibitor fidarestat prevents retinal oxidative stress and vascular endothelial growth factor overexpression in streptozotocin-diabetic rats. Diabetes 52: 864–871. 12606532 10.2337/diabetes.52.3.864

[37] Drel VR, Pacher P, Ali TK, Shin J, Julius U, et al (2008) Aldose reductase inhibitor fidarestat counteracts diabetes-associated cataract formation, retinal oxidative-nitrosative stress, glial activation, and apoptosis. Int J Mol Med 21: 667–676. 18506358 PMC2527815

[38] Gerhardinger C, Dagher Z, Sebastiani P, Park YS, Lorenzi M (2009) The transforming growth factor-beta pathway is a common target of drugs that prevent experimental diabetic retinopathy. Diabetes 58: 1659–1667. 19401417 10.2337/db08-1008 PMC2699853

[39] Hattori T, Matsubara A, Taniguchi K, Ogura Y (2010) Aldose reductase inhibitor fidarestat attenuates leukocyte-endothelial interactions in experimental diabetic rat retina in vivo. Curr Eye Res 35: 146–154. 20136425 10.3109/02713680903447918

[40] Ramana KV, Srivastava SK (2006) Mediation of aldose reductase in lipopolysaccharide-induced inflammatory signals in mouse peritoneal macrophages. Cytokine 36: 115–122. 17174561 10.1016/j.cyto.2006.11.003 PMC1850149

[41] Shoeb M, Yadav UC, Srivastava SK, Ramana KV (2011) Inhibition of aldose reductase prevents endotoxin-induced inflammation by regulating the arachidonic acid pathway in murine macrophages. Free Radic Biol Med 51: 1686–1696. 21856412 10.1016/j.freeradbiomed.2011.07.024 PMC3188329

[42] Pandey S, Srivastava SK, Ramana KV (2012) A potential therapeutic role for aldose reductase inhibitors in the treatment of endotoxin-related inflammatory diseases. Expert Opin Investig Drugs 21: 329–339. 10.1517/13543784.2012.656198 PMC3315185 22283786

[43] Abdillahi M, Ananthakrishnan R, Vedantham S, Shang L, Zhu Z, et al (2012) Aldose reductase modulates cardiac glycogen synthase kinase-3beta phosphorylation during ischemia-reperfusion. Am J Physiol Heart Circ Physiol 303: H297–308. 22661511 10.1152/ajpheart.00999.2011 PMC3423166

[44] Spite M, Baba SP, Ahmed Y, Barski OA, Nijhawan K, et al (2007) Substrate specificity and catalytic efficiency of aldo-keto reductases with phospholipid aldehydes. Biochem J 405: 95–105. 17381426 10.1042/BJ20061743 PMC1925154

[45] Ruiz FX, Gallego O, Ardevol A, Moro A, Dominguez M, et al (2009) Aldo-keto reductases from the AKR1B subfamily: retinoid specificity and control of cellular retinoic acid levels. Chem Biol Interact 178: 171–177. 19014918 10.1016/j.cbi.2008.10.027

[46] Cheung AK, Fung MK, Lo AC, Lam TT, So KF, et al (2005) Aldose reductase deficiency prevents diabetes-induced blood-retinal barrier breakdown, apoptosis, and glial reactivation in the retina of db/db mice. Diabetes 54: 3119–3125. 16249434 10.2337/diabetes.54.11.3119

[47] MacGregor LC, Matschinsky FM (1985) Treatment with aldose reductase inhibitor or with myo-inositol arrests deterioration of the electroretinogram of diabetic rats. J Clin Invest 76: 887–889. 3928685 10.1172/JCI112048 PMC423926

[48] Hotta N, Koh N, Sakakibara F, Nakamura J, Hamada Y, et al (1995) An aldose reductase inhibitor, TAT, prevents electroretinographic abnormalities and ADP-induced hyperaggregability in streptozotocin-induced diabetic rats. Eur J Clin Invest 25: 948–954. 8719936 10.1111/j.1365-2362.1995.tb01972.x

[49] Hotta N, Koh N, Sakakibara F, Nakamura J, Hara T, et al (1997) Effect of an aldose reductase inhibitor on abnormalities of electroretinogram and vascular factors in diabetic rats. Eur J Pharmacol 326: 45–51. 9178654 10.1016/s0014-2999(97)00135-0

[50] Samuels IS, Lee CA, Petrash JM, Peachey NS, Kern TS (2012) Exclusion of Aldose Reductase as a mediator of ERG deficits in a mouse model of diabetic eye disease. Visual Neuroscience In press. 10.1017/S0952523812000326 PMC3745719 23101909

[51] Joussen AM, Poulaki V, Le ML, Koizumi K, Esser C, et al (2004) A central role for inflammation in the pathogenesis of diabetic retinopathy. Faseb J 18: 1450–1452. 15231732 10.1096/fj.03-1476fje

[52] Zheng L, Du Y, Miller C, Gubitosi-Klug RA, Ball S, et al (2007) Critical role of inducible nitric oxide synthase in degeneration of retinal capillaries in mice with streptozotocin-induced diabetes. Diabetologia 50: 1987–1996. 17583794 10.1007/s00125-007-0734-9

[53] Du Y, Smith MA, Miller CM, Kern TS (2002) Diabetes-induced nitrative stress in the retina, and correction by aminoguanidine. J Neurochem 80: 771–779. 11948240 10.1046/j.0022-3042.2001.00737.x

[54] El-Remessy AB, Abou-Mohamed G, Caldwell RW, Caldwell RB (2003) High glucose-induced tyrosine nitration in endothelial cells: role of eNOS uncoupling and aldose reductase activation. Invest Ophthalmol Vis Sci 44: 3135–3143. 12824263 10.1167/iovs.02-1022

[55] Papezikova I, Pekarova M, Chatzopoulou M, Nicolaou I, Demopoulos V, et al (2008) The effect of aldose reductase inhibition by JMC-2004 on hyperglycemia-induced endothelial dysfunction. Neuro Endocrinol Lett 29: 775–778. 18987578

[56] Du Y, Tang J, Li G, Berti-Mattera L, Lee CA, et al (2010) Effects of p38 MAPK inhibition on early stages of diabetic retinopathy and sensory nerve function. Invest Ophthalmol Vis Sci 51: 2158–2164. 20071676 10.1167/iovs.09-3674 PMC2868413

